# Multi-luminance mobility testing after gene therapy in the context of retinal functional diagnostics

**DOI:** 10.1007/s00417-023-06237-4

**Published:** 2023-09-28

**Authors:** Ronja Jung, Melanie Kempf, Saskia Holocher, Friederike C. Kortüm, Krunoslav Stingl, Katarina Stingl

**Affiliations:** 1 University Eye Hospital, Center for Ophthalmology, University of Tuebingen, Elfriede-Aulhorn-Str.7, Tübingen, Germany; 2 Center for Rare Eye Diseases, University of Tübingen, Tübingen, Germany

**Keywords:** Gene therapy, Voretigene neparvovec, Mobility testing, Clinical endpoints

## Abstract

**Background:**

Voretigene neparvovec (Luxturna®) is the first approved gene therapy for RPE65-linked Leber congenital amaurosis (LCA). Though individual effects are highly variable, most recipients report improved vision in everyday life. To describe such effects, visual navigation tests are now frequently used in clinical trials. However, it is still unclear how their results should be interpreted compared to conventional parameters of visual function.

**Methods:**

Seven LCA patients underwent a multi-luminance visual navigation test (Ora-VNCTM) before and 3 months after receiving Luxturna gene therapy. Their performance was rated based on the luminance level at which they passed the course. Differences between the first and second test were correlated to changes in visual acuity, full-field stimulus thresholds, chromatic pupil campimetry, and dark-adapted perimetry.

**Results:**

A few patients displayed notable improvements in conventional measures of visual function whereas patients with advanced retinal degeneration showed no relevant changes. Independent of these results, almost all participants improved in the visual navigation task by one or more levels. The improvement in the mobility test was best correlated to the change in full-field stimulus thresholds. Other measures of visual functions showed no clear correlation with visual navigation.

**Discussion:**

In patients who passed the test’s more difficult levels, improved visual navigation can be attributed to the reactivation of rods. However, the performance of patients with low vision seemed to depend much more on confounding factors in the easier levels. In sum, such tests might only be meaningful for patients with better preserved visual functions.


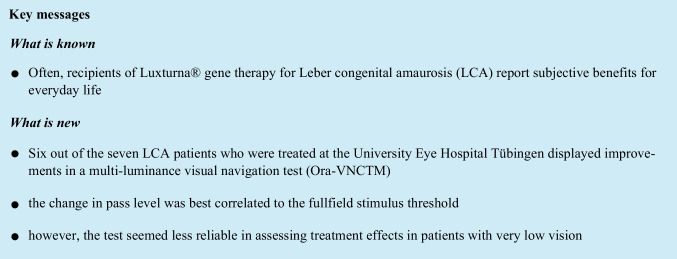


## Introduction

Leber congenital amaurosis (LCA) is a severe form of inherited retinal dystrophy. It is hallmarked by a rapidly progressing visual impairment which first presents in early childhood and usually leads to complete blindness by the fourth decade of life [1]. About 6% of all cases of LCA are caused by biallelic mutations in *RPE65* [4] which disrupt the recycling of rhodopsin [10]. Nonetheless, photoreceptor morphology may be preserved for several years [5], raising the possibility of functional recovery via gene supplementation.

In late 2017, the first gene therapy for *RPE65*-linked LCA (voretigene neparvovec, Luxturna®) became available in the USA. Studies have demonstrated that it may not only slow the progression of degeneration but also restore some visual function [6, 9, 12]. Yet, this improvement seems to be mostly limited to younger patients [13]. Nonetheless, many recipients report improved vision in everyday life. To assess this effect objectively, visual navigation tests are increasingly used as endpoints in clinical trials [11]. But as these tests rely on the patient’s active cooperation, their results can be susceptible to confounding factors such as motivation and learning. These factors could undermine the test’s utility for identifying treatment effects in patients with very low vision. Another limitation of the procedure is the inter-individual differences in the patients’ remaining visual abilities which usually requires a higher level of adaptability as compared to other tests.

To investigate the actual meaning of improvements in these tests, we did a multi-luminance mobility test with recipients of voretigene neparvovec and correlated the results with other (objective) clinical parameters of visual function which have already been used to demonstrate the effectiveness of the therapy.

The purpose of this paper is to provide fellow researchers who are currently looking for suitable read-out parameters for their clinical trials with exemplary data for mobility testing in patients with retinal degeneration and to point out a few practical considerations and limitations.

## Methods

### Participants

Seven patients with biallelic mutations in the *RPE65* gene were treated with voretigene neparvovec at the University Eye Hospital in Tuebingen between October 2019 and June 2020. Among them, four subjects were treated on one eye, and three on both eyes.

### Ophthalmological examination

All participants underwent thorough ophthalmological examinations before the injection (B), and 3 months (M3) later. The examinations included best corrected visual acuity (BCVA) testing using EDTRS charts and dark-adapted chromatic (DAC) perimetry (Medmont International Pty Ltd; Victoria, Australia) with shortened cyan (505 nm, 0 to − 75 dB, 0 dB corresponding to 17.6 cd/m^2^) and red (625 nm, 0 to − 50 dB) protocols [13, 14]. The thresholds were determined using a 4–2 staircase strategy, and the average sensitivity was calculated over all 36 tested points in the central 30° visual field. The full-field light sensitivity threshold (FST) was assessed for red, white, and blue light stimulation using the Espion ColorDome™ LED full-field stimulator (Diagnosys LLC, Lowell, MA) with a reference intensity (0 dB) of 0.01 cd s/m^2^. Local rod and cone function was evaluated by means of chromatic pupil campimetry (CPC) as described in recent papers [7, 13, 14]. In short, CPC quantifies the retinal function based on the relative pupillary constriction evoked by a blue or red stimulus in the corresponding area in the visual field. For further analysis, we calculated the average amplitudes over all tested locations.

### Visual navigation task

The patient’s navigational skills were evaluated using the multi-luminance obstacle course provided by Ora® (Ora-VNC™, Ora, Inc., Andover, MA, USA). The test has been specifically designed for interventional trials for inherited retinal degenerative diseases [3]. It provides a standardized parameter for the participant’s visual navigation based on his ability to walk along a highlighted path while avoiding different obstacles along the way. The tiles that form the path can be assembled into different layouts, each covering an area of approximately 5 by 7 m^2^. The procedure can be performed at low- and high-contrast conditions (called LCVNC and HCVNC, respectively), the latter of which uses brighter tiles, simpler layouts, and bigger obstacles for patients in later stages of retinal degeneration. The courses are performed at varying illuminances in the range between 0.35 and 500 Lux, corresponding to different levels of difficulty.

At the baseline visit, the subject completed a short training on the LCVNC at the highest light intensity (500 Lux) to determine the appropriate contrast for the actual baseline test. If the subject passed the test, the LCVNC was used, if not, the HCVNC was used. After 20 min of dark adaptation, the first course was performed at the lowest illuminance (0.35 Lux) on a new layout and with the untreated eye patched. The light intensity was increased stepwise (1, 3, 8, 22, 63, 178 Lux) till the subject passed a course, up to a maximum of 500 Lux. Passing was defined as reaching the final tile within the 5-min time limit without stepping completely off the path or exceeding the allowed maximum number of errors (five in the LCVNC, three in the HCVNC). The layout was replaced with each new attempt to minimize learning effects. At the beginning of a new trial, the patients were led to the start of the path and asked if they were able to see the path and could do the test at the given light level.

### Statistics

The main outcome measure of the VNC was the level of difficulty (ranging from 1 to 8) at which the subject was passed the course, corresponding to light levels. Differences between the pass levels at baseline and follow-up were assessed and correlated to changes in the BCVA, average CPC amplitudes, average DAC thresholds, and FST values. Additionally, corresponding correlations were calculated separately for baseline and follow-up measurements.

The statistical analysis was performed in MATLAB (R2019a, The MathWorks, Inc., Natick, MA, USA). Spearman’s correlation coefficients were used to calculate the relations between the outcome measures across individuals.

## Results

Two patients (P3 and P8) passed the test with 500 Lux LCVNC at screening and thus performed the low-contrast version. The remaining subjects did not pass and were tested with the HCVNC. The median pass level before treatment was 63 Lux. The lowest luminance level at which participants were able to complete the course was 8 Lux. Most participants passed at 178 Lux or below. One subject (P12, left eye) required the highest light level (500 Lux) in the HCVNC at baseline, which differed from the performance of the remaining participants by more than two standard deviations and was therefore excluded from the correlation analysis.

Three months after treatment, all but one participant improved by at least one level, with a median of 2 levels. In the LCVNC, P3 improved by two levels and P8 by one level. One participant passed the HCVNC (P1, LE only) at the lowest luminance level (0.35 Lux), corresponding to an improvement by three levels. Only P13 passed at a higher level after treatment (63 vs 178 Lux).

### Scotopic tests

**Fig. 1:**
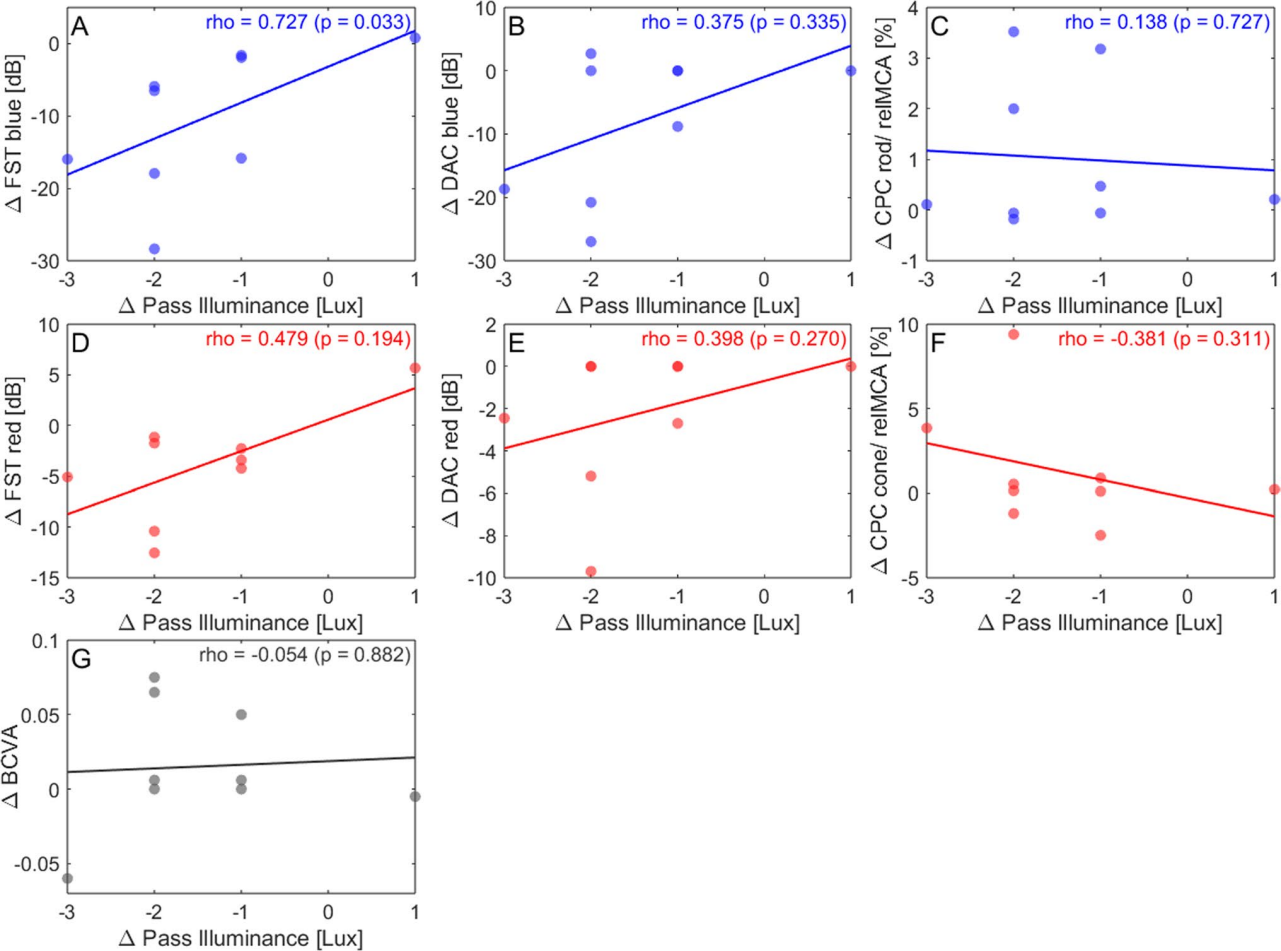
Correlation analysis. The correlation between parameters of visual function (*y*-axis) and the pass level of the visual navigation course (*x*-axis) was analyzed, using the difference between the measurements at baseline and three months after treatment (M3-B). Solid lines represent the linear fit. Scotopic tests include FST with blue (**A**) and red (**D**) light (in dB), DAC with cyan (**B**) and red (**E**) light (in dB), and (**C**) the pupil constriction amplitudes in response to blue light stimuli measured via CPC. Photopic measurements include (**F**) CPC constriction amplitudes with red light stimuli and (**G**) visual acuity. BCVA, best-corrected visual acuity; CPC, chromatic pupil campimetry; DAC, dark-adapted chromatic perimeter; FST, full-field stimulus threshold; relMCA, relative maximal constriction amplitude. Created in Matlab

**Fig. 2:**
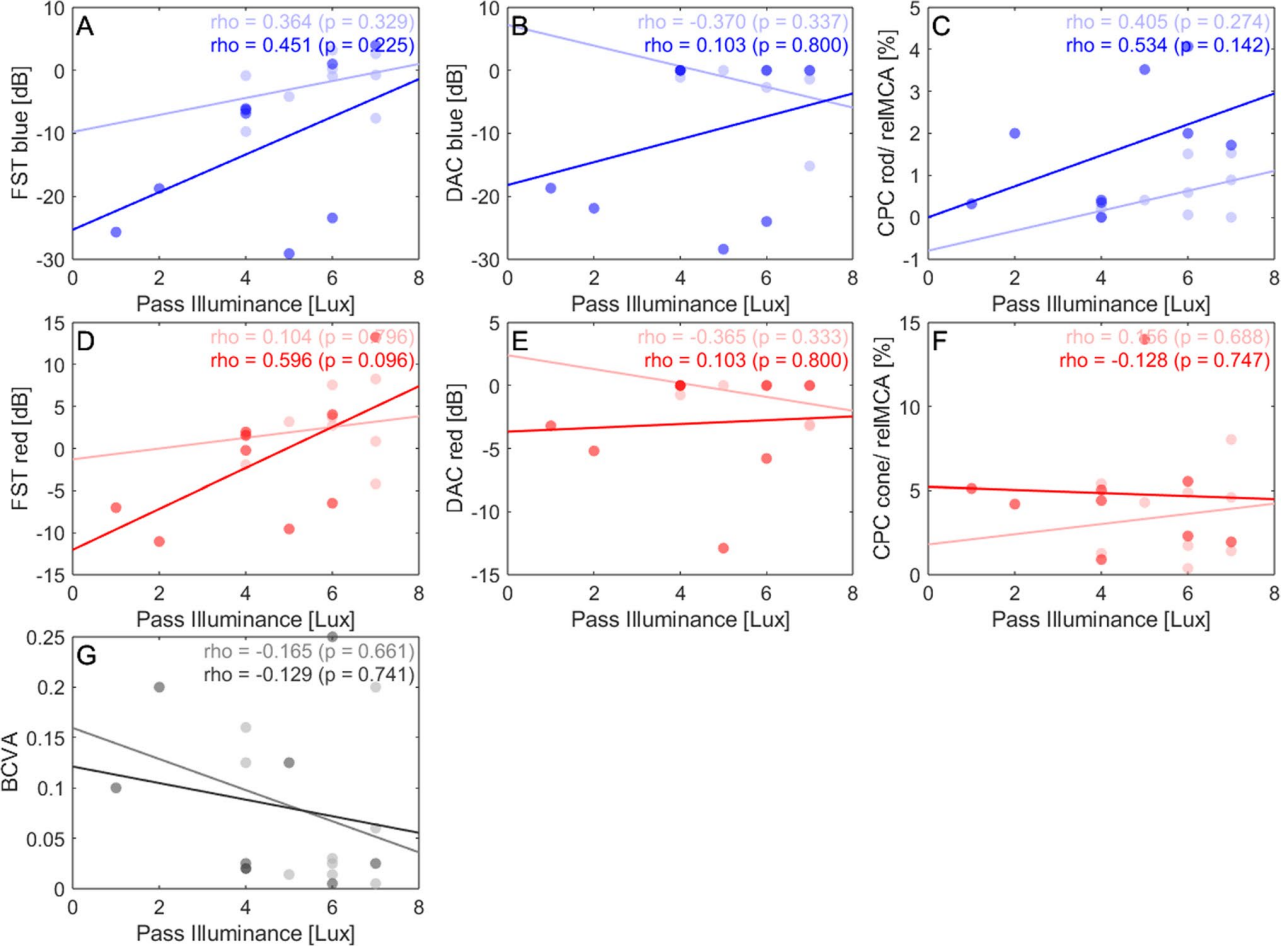
Correlation analysis before vs 3 months after treatment. Dark red, blue, and gray scatter plots represent the measurements after treatment. Light scatter plots represent baseline measurements. Scotopic parameters include FST with blue (**A**) and red (**D**) light (in dB), DAC with cyan (**B**) and red (**E**) light (in dB), and (**C**) CPC with blue light stimuli, represented by the relative maximum pupil constriction amplitude. Photopic parameters include (**F**) CPC with red light stimuli and (**G**) visual acuity. BCVA, best-corrected visual acuity; CPC, chromatic pupil campimetry; DAC, dark-adapted chromatic perimeter; FST, full-field stimulus threshold; relMCA, relative maximal constriction amplitude. Created in Matlab

Rod function was assessed by multiple measures, including CPC with blue light, FST with blue and red light, and averaged DAC thresholds for cyan and red stimuli, respectively. Within 3 months after treatment, the FST values for blue light improved on average by − 9.22 ± 9.82 dB (*p* = 0.016, *t*-stat = 2.968). The FST for red light improved by − 3.81 ± 5.02 dB (*p* = 0.040, *t*-stat = 2.399). The improvement in the VNC pass level was correlated (Fig. 1A and D) with the change in the FST with blue (*p* = 0.033, rho = 0.727), as to a lesser extent also with red light stimuli (*p* = 0.194, rho = 0.479). Similarly, there was a moderate correlation between the individual post-treatment FST measurements with red light and pass levels (*p* = 0.096, rho = 0.596; Fig. 2D). P13, who did not improve in the VNC, was also the only patient with slightly increased thresholds in both tests after treatment.

Three participants displayed noticeable improvements in DAC perimetry, ranging from − 8.80 to − 27.00 dB for blue and from − 2.45 to − 9.70 dB for red. The remaining patients were unable to detect any lights in the DAC even at the highest tested intensity before and after treatment. Accordingly, there was no correlation with the results of the VNC.

In the CPC, no participant had pupillary responses to blue light stimuli at baseline. After treatment, small responses were measurable in P3 and P8, who had passed the LCVNC.

### Correlates of cone function

Cone function was assessed via BCVA and photopic CPC. The visual acuity improved slightly in four patients, most notably in P3 (0.06 before vs 0.125 after therapy). Only five participants had residual cone-mediated pupillary responses to red light stimulation at baseline. Among those, two (P1 and P3) displayed a noticeable increase in the average relMCA at follow-up (+ 3.85% and + 9.40%). In addition, new cone responses could be measured in P12 at the 3 M visit in the treated area of the retina, indicating a recovery of cone function due to the gene therapy.

Yet, there were no significant correlations to the change in VNC pass levels, neither with visual acuity (*p* = 0.882, rho =  − 0.054; Fig. 1G), nor with CPC (*p* = 0.311, rho =  − 0.381; Fig. 1F). The same applies to the absolute values before and after injection (Fig. 2F and G).

## Discussion

Three-dimensional visual navigation tests have been used in several studies to evaluate the impact of treatments for retinal degenerative diseases [2, 3, 8]. However, depending on the targeted patient population, these tests may have certain limitations that could affect its applicability as a clinical read-out parameter.

Here, we investigated the dependencies between conventional parameters of visual function and the performance in a mobility test to evaluate its usefulness for identifying improvements in vision following gene therapy with voretigene neparvovec.

A few recipients displayed notable improvements in multiple parameters of visual function after therapy. Specifically, reductions in FST and DAC thresholds (representing rod sensitivity), as well as improved pupil responses (representing the neurons’ sum activation), point to a local reactivation of rods in the treated area in a few patients [13]. Subjects with advanced retinal degeneration, however, often showed no measurable effects on standard parameters.

Since rod vision contributes strongly to orientation at lower luminances, participants with measurable changes in scotopic vision were expected to show stronger changes in the VNC compared to non-responders. Indeed, the correlation between the improvement in the VNC and the change in FST with blue light indicates that improved performance in the VNC is driven by the reactivation of rods. However, almost all subjects improved by one or even two light levels, regardless of their results in other tests. Furthermore, there was no significant correlation with the absolute FST values from the first and second measurement. Hence, individual improvements in the VNC seemingly do not accurately reflect treatment effects in all patients. There are several factors that may explain this apparent lack of specificity.

In general, it needs to be considered that standard ophthalmological procedures measure very specific aspects of rod or cone vision, whereas tests for visual navigation rely on higher visual processes. Hence, their results may not be easily comparable with one another.

But beyond that, the lack of correlation could also be attributed to our data analysis. Specifically, because of the small size of our patient sample, we included all treated eyes as separate samples, which could have compromised the independence of the data and altered the outcome.

Furthermore, some improvements in the VNC of patients with no measurable change in other measures may be explained by learning effects. Although the layout of the course was replaced after each attempt, some subjects still learned to avoid mistakes after a few trials (e.g., by walking slowly and pausing more often to scan the environment). In accordance with this, Cideciyan et al. [3], who used the Ora-VNC™ in a trial for oligonucleotide therapy for LCA, found that improvements were largely symmetrical between treated and untreated eyes. On the other hand, their setup also deviated slightly from the recommended procedure. Specifically, they used a smaller layout, and their light levels were not spaced logarithmically. In combination, these deviations may have reduced the VNC’s repeatability. Similarly, we only used a simplified version of the original Ora-VNC™ protocol for this study that included less randomizations to reduce the time needed for the procedure. In the original version, the participant should complete all levels of both the high- and low-contrast course at screening, which in turn has to be repeated for each eye. Though this procedure may prevent learning effects in the course of follow-up visits, a major disadvantage is that it takes many hours to complete an entire screening test, and follow-up measurements still often take a few hours depending on the subject’s performance.

Another aspect which may undermine the test’s outcome is the low difficulty of the high-contrast test variation, designed for patients with very low vision. Specifically, most of the obstacles in the HCVNC are positioned next to the main path and do not necessarily have to be seen in order to be evaded as long as the participant stays in the middle of the path. Consequently, it was difficult to determine if the subject passed because he actually recognized the obstacles or simply by chance. Only two RP patients (P3, P8) in the present study were able to pass the LCVNC at screening. Both showed similar improvements in FST and DAC thresholds, indicating a reactivation of rods. Despite that, they did not display stronger improvements in VNC levels than other patients (though postoperative foveal scarring could have interfered with P8’s visual navigation [13]). In sum, the change in pass levels of subjects who completed the LCVNC is not comparable with those who did the HCVNC. As to account for this difference between test versions, Cideciyan et al. [3] did not evaluate the absolute luminance levels but instead used a continuous scale from the darkest level of the LCVNC to the brightest level of the HCVNC. Nonetheless, they found no significant differences between treated and untreated eyes (potentially due to the previously mentioned deviations from the original protocol). Other studies on visual navigation assessed the time that their participants needed to complete the obstacle course, showing that RP patients and healthy controls can best be distinguished based on their preferred walking speed [8]. However, our own experience was that speed mostly depends on the subject’s approach to the test,i.e., while some patients proceeded very cautiously to avoid errors as much as possible, others would just walk along the path at their normal walking speed to reach the end of the course as fast as possible, even at the risk of hitting more obstacles.

Related to that, another issue is that the course requires more active participation than other procedures. Appropriately, performance can be biased more easily by fatigue or motivation. P13, for example, whose pass level had increased after treatment, became more reluctant to do the test after initial failures at lower luminances and asked to skip levels.

In summary, the results of the VNC are not as easy to interpret as conventional measures of vision. Especially in the late disease stages, it is difficult to assess whether improvements are caused by the treatment or by subjective factors. To be used as a clinical endpoint, both the outcome parameters and the specific details of the testing procedure should be chosen carefully depending on the patients’ stage of retinal degeneration.

## Conclusion

Following treatment with voretigene neparvovec®, most patients showed improvements in visual navigation. This change was correlated with the improvement in FST with blue light stimuli, but other clinical parameters of visual functions, including DAC, CPC, and BCVA, were largely uncorrelated to the pass level. The results indicate rod sensitivity as a main predictor for the performance in the VNC. However, patients with no measurable improvements in standard tests also usually improved in the VNC. This lack of specificity may have been caused by several factors, including learning effects and poor comparability between VNC versions. To achieve higher reliability in the VNC, several points regarding the implementation and interpretation should be considered: Firstly, future trials should include training prior to the first measurement so as to minimize learning effects. Secondly, both the treated and untreated eye should be measured to control for subjective influencing factors. On the other hand, the reliability may be compromised if the complete protocol cannot be performed due to spatial or time limitations. Unfortunately, the inclusion of all these steps makes the test even more time-consuming. Finally, if patients in different stages of retinal degeneration complete very different versions of the mobility test, their improvements may no longer be comparable with one another and should be analyzed separately.

## References

[1] Chao DL, Burr A, Pennesi M (1993) GeneReviews®. RPE65-related Leber congenital amaurosis/early-onset severe retinal dystrophy. In: Adam MP, Ardinger HH, Pagon RA, Wallace SE, Bean LJH, Mirzaa G, Amemiya A (eds) Seattle (WA) 31725251

[2] Chung DC, McCague S, Yu Z-F, Thill S, DiStefano-Pappas J, Bennett J (2018). Novel mobility test to assess functional vision in patients with inherited retinal dystrophies. Clin Exp Ophthalmol.

[3] Cideciyan AV, Jacobson SG, Drack AV, Ho AC, Charng J, Garafalo AV (2019). Effect of an intravitreal antisense oligonucleotide on vision in Leber congenital amaurosis due to a photoreceptor cilium defect. Nat Med.

[4] den Hollander AI, Roepman R, Koenekoop RK, Cremers FPM (2008). Leber congenital amaurosis. Genes, proteins and disease mechanisms. Prog Retin Eye Res.

[5] Jacobson SG, Aleman TS, Cideciyan AV, Sumaroka A, Schwartz SB, Windsor EAM (2005). Identifying photoreceptors in blind eyes caused by RPE65 mutations. Prerequisite for human gene therapy success. Proc Natl Acad Sci U S A.

[6] Jacobson SG, Cideciyan AV, Ratnakaram R, Heon E, Schwartz SB, Roman AJ (2012). Gene therapy for Leber congenital amaurosis caused by RPE65 mutations. Safety and efficacy in 15 children and adults followed up to 3 years. Arch Ophthalmol.

[7] Kelbsch C, Stingl K, Kempf M, Strasser T, Jung R, Kuehlewein L (2019). Objective measurement of local rod and cone function using gaze-controlled chromatic pupil campimetry in healthy subjects. Transl Vis Sci Technol.

[8] Kumaran N, Ali RR, Tyler NA, Bainbridge JWB, Michaelides M, Rubin GS (2020). Validation of a vision-guided mobility assessment for RPE65-associated retinal dystrophy. Transl Vis Sci Technol.

[9] Maguire AM, High KA, Auricchio A, Wright JF, Pierce EA, Testa F (2009). Age-dependent effects of RPE65 gene therapy for Leber’s congenital amaurosis. A phase 1 dose-escalation trial. Lancet.

[10] Redmond TM, Yu S, Lee E, Bok D, Hamasaki D, Chen N (1998). Rpe65 is necessary for production of 11-cis-vitamin A in the retinal visual cycle. Nat Genet.

[11] Russell S, Bennett J, Wellman JA, Chung DC, Yu Z-F, Tillman A (2017). Efficacy and safety of voretigene neparvovec (AAV2-hRPE65v2) in patients with RPE65-mediated inherited retinal dystrophy. A randomised, controlled, open-label, phase 3 trial. Lancet.

[12] Simonelli F, Maguire AM, Testa F, Pierce EA, Mingozzi F, Bennicelli JL (2010). Gene therapy for Leber’s congenital amaurosis is safe and effective through 1.5 years after vector administration. Mol Ther.

[13] Stingl K, Kempf M, Bartz-Schmidt KU, Dimopoulos S, Reichel F, Jung R (2021). Spatial and temporal resolution of the photoreceptors rescue dynamics after treatment with voretigene neparvovec. Br J Ophthalmol.

[14] Stingl K, Stingl K, Nowomiejska K, Kuehlewein L, Kohl S, Kempf M (2020). Clinical protocols for the evaluation of rod function. Ophthalmologica.

